# Acceptability of receiving lifestyle advice at cervical, breast and bowel cancer screening

**DOI:** 10.1016/j.ypmed.2018.12.005

**Published:** 2019-03

**Authors:** Claire Stevens, Charlotte Vrinten, Samuel G. Smith, Jo Waller, Rebecca J. Beeken

**Affiliations:** a Department of Behavioural Science and Health, University College London, London WC1E 6BT, UK; b Leeds Institute of Health Sciences, University of Leeds, Leeds LS2 9NL, UK

**Keywords:** Cancer screening, Teachable moment, Lifestyle, Cancer prevention, Behaviour change

## Abstract

Cancer screening could be an opportunity to deliver cancer prevention advice, but it is not known how such information would be received. We explored willingness to receive lifestyle advice in the context of the English National Health Service cervical, breast, and bowel (FS; flexible sigmoidoscopy) screening programmes. A population-based survey was conducted in 2016 to collect nationally representative data on willingness to receive lifestyle advice across cervical (*n* = 768), breast (*n* = 420) and FS (*n* = 308) screening programmes. Additional items assessed the impact of lifestyle advice on screening attendance, preference for receiving advice in the event of an abnormal screening result, and timing of advice. Most respondents were willing to receive lifestyle advice around the time of cancer screening (cervical 78.9%, breast 79.4%, FS 81.8%), and if their results were abnormal (cervical 86.3%, breast 83.0%, FS 85.1%). A small proportion indicated it may discourage future attendance (cervical 4.9%, breast 7.0%, FS 8.8%). Most preferred information to be delivered at the screening appointment (cervical 69.8%, breast 72.6%, FS 70.7%). There were no associations between sociodemographic characteristics and willingness to receive lifestyle advice at breast screening. For those intending to attend cervical screening, non-White ethnicity and higher education were associated with increased willingness to receive lifestyle advice. Women were more likely to be willing to receive advice at FS screening than men. Providing lifestyle advice at cancer screening is likely to be acceptable to the general population. The optimal approach for delivery needs careful consideration to minimise potential negative effects on screening attendance.

## Highlights

• The majority of people are willing to receive lifestyle advice at cancer screening
• Most people want this advice to be delivered at the screening appointment itself
• Some people may be deterred from attending cancer screening if advice is given

## 1 Introduction

In 2014 there were 350,000 cancer diagnoses in the UK, and by 2035 annual diagnoses are expected to exceed 500,000 (Cancer Research, 2016; Smittenaar et al., 2016). The link between lifestyle and the development of many common cancers is well established (Brown et al., 2018). Tobacco use is the single greatest cancer risk factor, however, the contribution of risk factors varies by cancer type. For example, the greatest risk factors for colorectal cancer include overweight, dietary factors, alcohol and tobacco use (Brown et al., 2018). Consequently, the importance of behavioural cancer prevention strategies is recognised (The Independent Cancer Taskforce, 2015).

Cancer screening has been described as a ‘teachable moment’, providing an opportunity to deliver cancer prevention advice and interventions (Senore et al., 2012). Cancer screening and risk factor reduction both impact cancer mortality (Edwards et al., 2010). Combined the two approaches are likely to have the greatest effects (Joshu et al., 2012). Providing lifestyle advice alongside cancer screening is consistent with English policy to ‘Make Every Contact Count’ by utilising interactions with the public to support health and wellbeing (Public Health England, 2016a). However, there is little evidence that cancer prevention advice is delivered routinely alongside cancer screening in the UK (Anderson et al., 2013).

Recent evidence suggests interventions can be delivered alongside cancer screening (Anderson et al., 2013; Senore et al., 2012). Interventions delivered at breast screening have promoted weight loss (Anderson et al., 2014b; Friedenreich et al., 2011). Low-intensity interventions delivered alongside bowel screening (FS; flexible sigmoidoscopy) have increased reported fruit and vegetable consumption within a screening trial setting (Baker and Wardle, 2002; Robb et al., 2010). At cervical screening, interventions targeting motivation to quit smoking and smoking cessation have produced mixed results (Chellini et al., 2012; Gorini et al., 2012; Hall et al., 2003; McBride et al., 1999).

There is concern that delivering information and interventions alongside screening could compromise uptake. Screening uptake varies within England, with FS uptake (43%) considerably lower than breast (71%), and cervical screening (74%) (Health and Social Care Information Centre, 2016a, Health and Social Care Information Centre, 2016b; McGregor et al., 2016). There is a socioeconomic gradient in screening participation, whereby more deprived populations are less likely to attend than less deprived populations (Douglas et al., 2016; McGregor et al., 2016; von Wagner et al., 2011). There are also associations between ethnicity and screening attendance, with Ethnic minority groups less likely to participate (McGregor et al., 2016; Moser et al., 2009; von Wagner et al., 2011). It is therefore important to explore sociodemographic determinants of interest in advice at cancer screening and anticipated changes to screening behaviour if lifestyle advice were routinely offered in NHS (National Health Service) Screening Programmes.

The acceptability of information delivered at population-based screening has been explored within the context of breast and cervical screening. A study of women attending mammography found 85% reported interest in receiving information about diet and exercise at breast screening clinics, and that this information was unlikely to impact future participation (Fisher et al., 2007). Similarly, a qualitative study of women who had attended breast screening reported most women were positive about receiving information about reducing body fatness, alcohol consumption and physical activity at screening (Conway et al., 2016). One study trialled the delivery of a magazine designed to provide information about lifestyle and cancer prevention to women attending breast screening clinics (Macleod and Anderson, 2018). Uptake was high among women who were actively offered the magazine (95%). Smoking cessation advice appears to be acceptable when delivered at cervical screening (Hall et al., 2007); most participants still intended to attend subsequent cervical screening appointments.

Using a population representative sample of English adults, this study used hypothetical scenarios to explore willingness to receive lifestyle advice alongside cervical, breast, and FS screening. These three screening modalities were selected as they involve interaction between patients and healthcare professionals, which has been suggested as important in the teachable moment (Lawson and Flocke, 2009). This research also sought to understand whether willingness to receive information around the time of screening differs according to the type of screening result received. We also investigated anticipated future screening behaviour if lifestyle advice were offered, and sociodemographic correlates of willingness to receive information. Finally, this research aimed to identify the preferred timing of advice during the screening process.

## 2 Methods

### 2.1 Design

Data were collected as part of a cross-sectional population-representative survey on the determinants of early detection and prevention behaviours related to cancer. Face-to-face computer-assisted interviews were conducted as part of an omnibus survey run by market research agency Taylor Nelson Sofres (TNS) in April and May 2016. Ethical approval was granted by the University College London Research Ethics Committee (Ref: 5771/002). Verbal consent was obtained at the start of interviews.

### 2.2 Participants

Random location sampling using 2011 Census data and Postcode Address File data was used to identify participants. Quotas were set for demographic characteristics to ensure a nationally representative sample. Questions relating to lifestyle advice at cancer screening were limited to three sub-samples. In line with current screening guidelines in England, women aged 25–64 (*n* = 768) were asked questions about cervical screening and women aged 47–70 were asked questions about breast screening (*n* = 420). Questions about bowel scope screening were asked of men and women aged 45–54 (*n* = 308). In England, people are invited to a one-off bowel scope screening appointment at the age of 55. So that intention to attend screening and the impact of advice on future screening attendance could be measured, questions relating to bowel scope screening were only asked of people approaching screening age.

### 2.3 Measures^1^

#### 2.3.1 Sociodemographic variables

Data were collected for age, gender, ethnicity and educational attainment (as a marker of social position). Ethnicity was categorised into White (including participants who identify as White British, White Irish and Other White groups) and non-White, based on UK Census ethnicity classifications. Education was measured using the item ‘*what is the highest level of educational qualification you have obtained*’, with responses categorised into ‘degree level or above’ (for people who have obtained an undergraduate bachelor's degree or above) and ‘education below degree level’.

#### 2.3.2 Cancer screening intention

Intention to participate in cancer screening was asked separately for the three programmes. Before answering questions about each screening modality, participants were shown a written and pictorial description of the screening programme. For cervical screening, women were asked *‘Will you go for cervical screening next time you are invited?’.* For breast screening, women were asked *‘Will you go for breast screening when, or next time you are invited?’.* For FS screening, people were asked ‘*Would you take up the offer for Bowel Scope screening if you were invited?’.* Four response options were offered (*Yes, definitely; Yes, probably; No, probably not; No, definitely not),* dichotomised into yes and no. Participants who did not intend to attend cancer screening were excluded from further analyses.

#### 2.3.3 Willingness to receive lifestyle advice at cancer screening

For those intending to attend any of the screening programmes, willingness to receive lifestyle advice was measured using three versions of the item ‘*Would you be willing to receive advice about making healthy lifestyle changes (for example, diet or physical activity) as part of the cervical/breast/bowel screening programme?’.* Five response options were offered, which categorised people as willing (*Yes, definitely; Yes, probably)*, or not *(No, probably not; No, definitely not; Not sure).* Responses were dichotomised as few participants selected the three latter response options. For each screening programme, an additional question assessed interest in lifestyle advice in the event of a screening result which required further investigations; *‘Would you be willing to receive lifestyle advice if your screening result suggested you needed to have further investigations?’.* The same response options were used for this item.

#### 2.3.4 Impact of lifestyle advice on cancer screening participation

All participants eligible to attend any of the three screening programmes were asked; ‘*If you knew you would receive advice about lifestyle as part of the cervical/breast/bowel screening programme, would this affect your willingness to attend cervical/breast/bowel screening?*’. Three response options were provided (*Yes, I would be more willing to attend; Yes, I would be less willing to attend; No, it would not affect my willingness to attend*).

#### 2.3.5 Timing of lifestyle advice

Preferences for the timing of lifestyle advice were assessed among participants who were intending to attend screening and willing to receive lifestyle advice ‘*When would you prefer to receive lifestyle advice as part of the cervical/breast/bowel screening programme?’.* Five response options were provided: *at the same time as my screening appointment; with my screening results; 2–4 weeks after attending screening; 1–3 months after attending screening; >3 months after attending screening*.

Participants were shown questions relating to all of the screening programmes they were eligible for, meaning women were asked about up to three screening programmes, whereas men were asked about just one.

### 2.4 Analyses

Descriptive analyses explored willingness to receive information around the time of screening, the effect of information provision on screening uptake and timing preferences. Three McNemar's tests explored differences between interest in lifestyle advice around screening in general and interest in the event that further investigations were required. Three logistic regression models were conducted, simultaneously entering age, gender, ethnicity, and education to identify sociodemographic correlates of willingness to receive lifestyle advice at cervical, breast and bowel (FS) cancer screening. Weights were used to ensure population representativeness. These were calculated by market research company TNS and based on age, region, social grade and working status. Sample characteristics are presented unweighted and weighted. Univariate and bivariate analyses are presented weighted. Multivariate analyses are presented unweighted. Where significance testing is necessary for the interpretation of results an alpha level of 0.05 was used.

## 3 Results

### 3.1 Sample characteristics

A total of 1037 (weighted *N* = 1041) participants were included in the analyses (Table 1). The mean age of the analytic sample was 47.6 years (SD 12.1). Most were female (81.1%, *n* = 844), reflecting the screening modalities studied. The majority were white (86.7%, *n* = 898) and educated at below degree level (56.0%, *n* = 653). The cervical screening sample included 768 women aged 25–70 (weighted *n* = 739), the breast screening sample included 420 women aged 47–70 (weighted *n* = 430), and the FS screening sample included 308 men and women aged 45–54 (weighted *n* = 386).

**Table 1:** Demographic characteristics of the total analytic sample and sub-samples for the cervical, breast, and FS screening scenarios.

	Total analytic sample				Cervical screening sample				Breast screening sample				FS screening sample^a^			
	Unweighted		Weighted		Unweighted		Weighted		Unweighted		Weighted		Unweighted		Weighted	
	(*n* = 1037)		(n = 1041)		(n = 768)		(n = 739)		(n = 420)		(n = 430)		(n = 308)		(n = 386)	
	M	SD	M	SD	M	SD	M	SD	M	SD	M	SD	M	SD	M	SD
Age																
	46.7	13.0	47.6	12.1	42.8	11.7	43.9	11.5	59.1	7.1	58.2	7.1	49.7	2.7	49.7	2.7

	n	%	n	%	n	%	n	%	n	%	n	%	n	%	n	%
Gender																
Male	147	14.2	197	18.9	–	–	–	–	**–**	**–**	**–**	**–**	147	47.7	197	50.9
Female	890	85.8	844	81.1	–	–	–	–	**–**	**–**	**–**	**–**	161	52.3	189	49.1

Ethnicity^b^																
White	886	85.9	898	86.7	647	84.8	631	86	378	90.7	387	90.8	262	85.9	331	86.5
Non white	146	14.2	138	13.3	116	15.2	103	14	39	9.4	39	9.2	43	14.1	52	13.5

Education																
Degree level or above	294	29.9	337	34.0	245	33.6	265	37.7	85	21.7	111	27.5	68	23.1	102	27.6
Qualifications below bachelor's degree level	688	70.1	653	65.0	484	66.4	437	62.3	307	78.3	291	72.5	226	76.9	267	72.4

Intention to attend screening																
Intends	–	–	–	–	671	94.9	651	95.4	362	92.8	378	94.0	241	84.6	311	87.1
Does not intend	–	–	–	–	36	5.1	31	4.6	28	7.2	24	6	44	15.4	46	12.9

a Flexible sigmoidoscopy.

b Based on dichotomisation of UK census classifications.

### 3.2 Willingness to receive lifestyle advice at cancer screening

Intention to participate in the three cancer screening programmes was high (cervical 95.4%, *n* = 651; breast 94.0%, *n* = 378; FS 87.1%, *n* = 311). Of those intending to attend cervical screening, most were willing to receive lifestyle advice alongside the NHS cervical screening programme (78.9%, *n* = 512). However, a greater proportion of this group were willing to receive advice if they received an abnormal screening result (86.3%, *n* = 558; McNemar's χ2 22.0, df = 644, *p* < 0.001). Most women who intended to attend breast screening were willing to receive lifestyle advice alongside breast screening (79.4%, *n* = 300) (Table 2). A similar proportion of this group indicated they would be willing to receive advice if they received an abnormal screening result (83.0%, *n* = 262; McNemar's χ23.38, df = 374, *p* = 0.087). For those intending to attend FS, the majority (81.8%, *n* = 252) were willing to receive lifestyle advice alongside bowel cancer screening. A similar proportion of this group were willing to receive advice if they received an abnormal screening result (85.1%, *n* = 252; McNemar's χ2 2.63, df = 307, *p* = 0.143).

**Table 2:** Willingness to receive lifestyle advice in cervical, breast, and FS screening scenarios.

Willing to receive lifestyle advice at cancer screening^a^				Willing to receive lifestyle advice if further investigations are needed^a^				McNemars χ2	
	n	% (95% CI)	Dichotomised % (95% CI)		n	% (95% CI)	Dichotomised % (95% CI)	χ2	p
Cervical cancer screening (*n* = 649)									
Yes, definitely	368	56.8 (52.8–60.7)		Yes, definitely	401	62.1 (58.1–65.9)			
Yes, probably	144	22.1 (19.1–25.6)	78.9 (75.5–82.0)	Yes, probably	157	24.3 (21.0–27.8)	86.3 (83.4–88.8)	22.0	0.001
No, probably not	50	7.8 (5.9–10.2)		No, probably not	26	4.0 (2.8–5.8)			
No, definitely not	62	9.6 (7.4–12.2)		No, definitely not	29	4.4 (3.0–6.5)			
Not sure	25	3.8 (2.6–5.5)	21.1 (18.0–24.6)	Not sure	34	5.2 (3.8–7.2)	13.7 (11.2–16.6)		

Breast cancer screening (*n* = 377)									
Yes, definitely	214	56.7 (51.2–61.9)		Yes, definitely	226	60.7 (55.2–65.9)			
Yes, probably	86	22.7 (18.5–27.6)	79.4 (74.7–83.4)	Yes, probably	83	22.3 (18.1–27.1)	83.0 (78.5–86.7)	3.38	0.087
No, probably not	30	8.0 (5.5–11.4)		No, probably not	24	6.5 (4.3–9.9)			
No, definitely not	36	9.6 (6.9–13.4)		No, definitely not	27	7.3 (4.9–10.8)			
Not sure	11	3.0 (1.7–5.3)	20.6 (16.6–25.3)	Not sure	12	3.2 (1.8–5.4)	17.0 (13.3–21.5)		

FS screening (*n* = 307)^b^									
Yes, definitely	159	51.5 (44.9–58.1)		Yes, definitely	170	55.1 (48.5–61.6)			
Yes, probably	93	30.3 (24.5–36.8)	81.8 (76.1–86.3)	Yes, probably	92	30.0 (24.3–36.3)	85.1 (79.5–89.4)	2.63	0.143
No, probably not	23	7.5 (4.8–11.7)		No, probably not	21	6.9 (4.0–11.5)			
No, definitely not	26	8.6 (5.5–13.3)		No, definitely not	18	6.0 (3.4–10.1)			
Not sure	6	2.1 (0.9–4.7)	18.2 (13.7–23.9)	Not sure	6	2.1 (0.9–4.7)	14.9 (10.7–20.5)		

a Data presented is weighted.

b Flexible sigmoidoscopy.

### 3.3 Sociodemographic correlates of willingness to receive lifestyle advice

Ethnicity and educational attainment were associated with willingness to receive advice at cervical screening (Table 3). Compared with white participants, non-white participants had greater odds of being willing to receive lifestyle advice (89.8% vs 77.0%; OR 2.39, 95% CI 1.16–4.93). Participants who reported education below degree level had lower odds of being willing to receive lifestyle advice at cervical screening when compared with participants who reported education at degree level or above (75.9% vs 87.0%; OR 0.52, 95%; CI 0.33–0.82). There were no associations between sociodemographic characteristics and willingness to receive lifestyle advice at breast screening. For FS screening, women had greater odds of reporting willingness than men (87.7% vs 74.8%; OR 2.35, 95% CI 1.17–4.75)^2^^,.^^3^

**Table 3:** Sociodemographic correlates of willingness to receive lifestyle advice in cervical, breast and FS screening scenarios (adjusted logistic regression models).

	Cervical screening sample (*n* = 637)^a^		Breast screening sample (*n* = 339)^a^		FS screening sample (*n* = 229)^a^, ^b^	
	OR	95% CI	OR	95% CI	OR	95% CI
Age	0.99	0.97–1.01	0.97	0.94–1.01	1.08	0.96–1.23

Gender						
Male	–	–	–	–	REF	–
Female	–	–	–	–	2.35	1.17–4.75

Ethnicity^c^						
White	REF	–	REF	–	REF	–
Non-white	2.39	1.16–4.93	2.33	0.68–7.99	1.04	0.36–2.98

Education						
Degree level or above	REF	–	REF	–	REF	–
Qualifications below bachelor's degree level	0.52	0.33–0.82	0.82	0.42–1.61	0.47	0.18–1.24

a Data is presented unweighted

b Flexible sigmoidoscopy

c Based on dichotomisation of UK census classifications

### 3.4 Impact of information provision on screening uptake

**Fig. 1:**
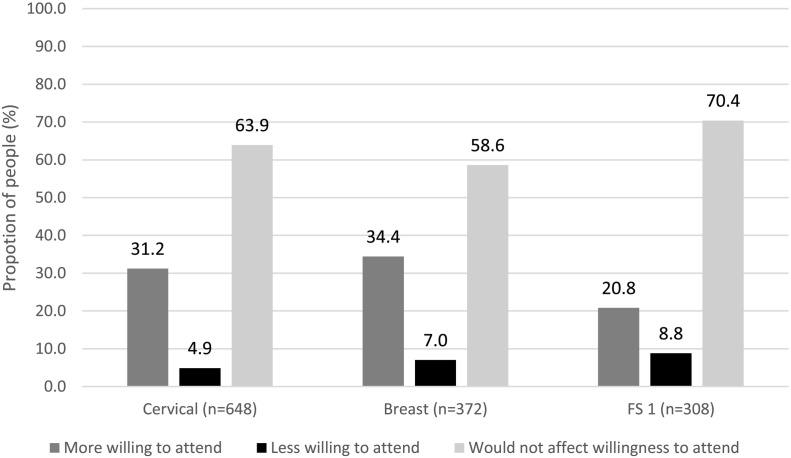
Impact of the provision of lifestyle advice on willingness to attend cancer screening, among participants who intend to attend their next cancer screening appointment. ^1^ Flexible sigmoidoscopy.

Across the three cancer screening programmes, the majority indicated the provision of lifestyle advice around the time of screening would not affect their willingness to attend (cervical 63.9%, *n* = 414; breast 58.6%, *n* = 218; FS 70.4%, *n* = 217) (Fig. 1). Some participants stated the provision of lifestyle advice would make them more willing to attend (cervical 31.2%, *n* = 202; breast 34.4%, *n* = 128; FS 20.8%, n = 64). However, for each of the screening programmes, a small minority of people felt the provision of advice would make them *less* willing to participate in future cancer screening (cervical 4.9%, *n* = 32; breast 7.0%, n = 26; FS 8.8%, *n* = 27).

### 3.5 Preferred timing of lifestyle advice at cancer screening

Most participants who were willing to receive lifestyle advice preferred this advice to be delivered at the screening appointment itself: cervical 69.8%, *n* = 353; breast 72.6%, *n* = 211; and FS screening 70.7%, *n* = 176, followed by with the screening results (cervical 21.2%, *n* = 107; breast 18.9%, n = 55; FS 17.4%, n = 43). Few participants wanted advice 2–4 weeks after attending screening (cervical 6.6%, n = 33; breast 6.9%, n = 20; FS 9.3%, *n* = 23), 1–3 months after attending (cervical 1.6%, n = 8; breast 1.1%, n = 3; FS 1.5%, n = 4), or more than three months after attending (cervical 0.9%, *n* = 4; breast 0.5%, n = 1; FS 1.1%, *n* = 3).

## 4 Discussion

In this large, population-based sample of English adults, the majority of people intending to attend NHS cancer screening programmes were willing to receive lifestyle advice, even if further investigations were required. For cervical screening, a greater proportion of participants were willing to receive advice when respondents considered it as part of a scenario where their results required further investigations. This effect was not observed for the breast and bowel screening samples, perhaps due to smaller sample sizes within these scenarios. A small proportion of people indicated they may be put off attending future screening appointments, suggesting screening uptake should be carefully monitored if lifestyle advice were routinely implemented. Among people willing to receive lifestyle advice, there was a strong preference for information to be delivered at the screening appointment.

The high proportion of people willing to receive lifestyle advice at cancer screening observed within our study (79–82%) is encouraging and comparable to previous findings (Fisher et al., 2007). It is unknown whether willingness to receive advice would remain high in real-life screening settings, and whether receipt of advice would result in behaviour change. Trials conducted within bowel and breast screening settings suggest around half of attendees (49% and 43% respectively) are interested in participating in interventions focused on topics such as physical activity, weight loss, and alcohol consumption (Anderson et al., 2014a; Anderson et al., 2014b). Retention of participants enrolled in these interventions appears to be high (93% and 81% respectively), suggesting it is feasible to deliver interventions within screening settings.

We identified sociodemographic factors associated with willingness to receive lifestyle advice at FS and cervical screening. For FS, women were more likely to express willingness to receive lifestyle advice compared with men. This is in line with previous research suggesting men are less likely to engage in health-promoting behaviours than women (Courtenay, 2000). Almost 90% of women were interested in lifestyle advice at FS, which may be higher than for cervical screening and should be confirmed in other samples. Education and ethnicity were associated with willingness to receive lifestyle advice at cervical screening. Higher education increased willingness to receive advice. The link between education and health behaviour is well established (Cutler and Lleras-Muney, 2010; Pampel et al., 2010). Except for alcohol consumption, unhealthy behaviours are more prevalent among populations of lower socioeconomic status (SES) (Stringhini et al., 2011). Within our sample, non-white women intending to attend cervical screening were more likely to be willing to receive advice than white women. However, previous research has found ethnic minority women less likely to participate in screening (Moser et al., 2009). Only a small proportion of our sample were non-white, therefore these results need to be interpreted cautiously and replicated in more ethnically diverse samples. Education and ethnicity were not associated with willingness to receive lifestyle advice at breast or bowel cancer screening, which may be a result of smaller sample sizes for these scenarios.

A sensitivity analysis reported in Supplementary File 1 explored the potential impact of current lifestyle on interest in lifestyle advice within the three scenarios. No associations were identified, however these analyses were limited by sample size due to missing data. A paper exploring interest in specific lifestyle advice topics (weight, physical activity, diet, smoking and alcohol consumption) found varying levels of interest in the different topics among people intending to attend cancer screening (Stevens et al., 2018). Within that sample, specific health behaviours were associated with interest in advice about the relevant lifestyle topic (e.g. those who were not physically active were more interested in receiving physical activity advice).

A small proportion of our sample felt that receipt of lifestyle advice around the time of screening would deter their future screening attendance. At a population level this could result in large numbers of people not receiving cancer screening. In 2015–2016, around 3 million women were tested as part of the NHS cervical screening programme, (Public Health England, 2016b). Within our sample, 5% of people reported lifestyle advice would make them less likely to attend cervical screening. This could equate to approximately 150,000 fewer women attending cervical screening. The proportion of people who indicated they would be deterred from attending cancer screening was small so it was not possible to explore sociodemographic associations. Future research should aim to confirm whether the provision of lifestyle advice at screening will exacerbate inequalities in screening uptake. If the provision of lifestyle advice is to be implemented alongside cancer screening, interventions must be designed to minimise the proportion of people deterred from attending screening. There would need to be strong evidence that the health benefits of any intervention off-sets the harm from any decrease in uptake.

Most participants indicated they would like to receive lifestyle advice at the screening appointment itself. Other research suggests the timing of interventions delivered in the context of cancer screening is important (McBride et al., 1999). It has also been reported that people attending screening would prefer advice to be given by an expert, such as a health professional (Fisher et al., 2007). This is in line with previous conceptualisations of the teachable moment as potentially reliant on interactions between patients and clinicians (Lawson and Flocke, 2009). Future work should, therefore, investigate how practicable it would be to deliver lifestyle advice within population cancer screening services, who would be best placed to deliver this advice, and how to join this up with patient preferences.

This research has limitations. It was not possible to obtain information about people who declined to participate in the survey. There may be differences between responders and non-responders. The proportion of people intending to attend screening, across the modalities, was higher than actual uptake rates. High cancer screening intentions are not unusual and overestimation of intention to perform a behaviour is known as the intention-behaviour gap (Sheeran, 2002). Within our sample, intention to attend FS was 87%, which is in line with intention rates reported in other English samples (Robb et al., 2008). However, actual FS uptake in England is around half of this figure (McGregor et al., 2016). Non-attenders were likely underrepresented making it difficult to draw conclusions about the effect of the provision of lifestyle advice on people who will, and people who will not attend screening. Additionally, while sociodemographic differences have been reported consistently for screening uptake, these differences may not be found when looking at screening intention (Robb et al., 2008). Therefore, this research may not accurately reflect sociodemographic differences in intentions or desire for lifestyle advice.

A further limitation is that this study was based on hypothetical scenarios around English cancer screening programmes. English cancer screening programmes are likely to differ from those offered in other countries, therefore these findings may not generalise to other populations. Scenarios presented in this research included attending cancer screening, receiving an abnormal screening result, and receiving lifestyle advice alongside screening. Hypothetical scenarios are likely to differ from appraisals of information delivered in a real-life screening setting. This may be a particular issue for FS screening, as this is a relatively new screening programme, which nobody in the sample would have been invited to participate in yet. Some participants will have answered questions relating to more than one screening programme, which may impact responses. This effect is difficult to determine because the number of programmes a person is eligible for is confounded by gender and age. This study is limited by the choice and wording of the measures used. We used education level as a proxy of SES, which may not best reflect a person's socioeconomic position. The use of dichotomised education and ethnicity variables also impact the interpretation of results. For example, we dichotomised education based on whether someone had attained education at degree level or above, which may have masked differences between groups educated below degree level. The wording of the questions may also have influenced responses. We used diet and physical activity as examples of lifestyle advice when asking about interest, different examples such as smoking cessation may have prompted a different response. Another limitation is that participants were only asked about their willingness to receive advice at breast, cervical and FS screening. Although interest in receiving advice was high across all three, it is not clear whether willingness to receive advice would be just as high in other settings, such as the workplace (Cahill and Lancaster, 2014). Finally, willingness to receive lifestyle advice at cancer screening may not translate into actual behaviour change. Further research is needed to understand adherence to lifestyle advice following its dissemination in a cancer screening setting, and to establish whether offering advice in this context is any more effective than giving it at other times.

## 5 Conclusion

This study was the first to investigate interest in lifestyle advice across three English cancer screening programmes. Interest was high, regardless of the outcome of a person's screening result. However, our results suggest a minority who would otherwise attend screening might be put off if lifestyle advice were offered. Future research should investigate the feasibility of providing lifestyle advice alongside cancer screening, and how best to deliver effective cancer risk reduction advice without compromising screening attendance.

## Financial support

CS is supported by a Cancer Research UK PhD Studentship (C416/A19488). CV is supported by a programme grant from Cancer Research UK awarded to Professor Jane Wardle (C1418/A14134). JW is funded by a Cancer Research UK career development fellowship (grant reference: C7492/A17219). SS is supported by Yorkshire Cancer Research University Academic Fellowship funding (L389SS). RJB is supported by Yorkshire Cancer Research University Academic Fellowship funding (L389RB).

## Conflicts of interest

The authors report no conflicts of interest.

## Appendix A Supplementary data

Supplementary material Image 1

## Footnotes

1 Cognitive interviews (*n* = 14) were used to assess the comprehension, clarity and acceptability of individual items. Finalised items were piloted online prior to inclusion in the final survey (*n* = 392).

2 We explored whether previous screening experience was associated with interest in lifestyle advice at breast at cervical screening. Adding past screening attendance did not change the direction of any correlates in either model, and past screening attendance was not associated with interest in advice in either model.

3 We explored whether participants' current lifestyle was associated with interest in lifestyle advice at cervical, breast and bowel screening. Participants provided self-report data on five cancer protective lifestyle factors (body mass index, fruit and vegetable consumption, smoking, physical activity, alcohol consumption). An index of these factors was not associated with interest in advice and results of the three models remained essentially unchanged. Further details can be found in Supplementary File 1.
