# Supplementary material for: Acceptability of receiving lifestyle advice at cervical, breast and bowel cancer screening
Source: Prev Med. 2019 Mar;120:19–25. doi: 10.1016/j.ypmed.2018.12.005 (PMC6380892; doi:10.1016/j.ypmed.2018.12.005)
Supplement: Supplementary file 1 — Supplementary material [file mmc1.docx]

**Supplementary file 1.** Exploring participant reported cancer protective factors as an additional correlate of interest in advice at cervical, breast and bowel cancer screening: a sensitivity analysis

The following sensitivity analysis explores participant reported cancer protective factors as a potential correlate of interest in advice at cervical, breast and bowel cancer screening. In line with previous research exploring behaviour change at cancer screening, an index of five cancer related lifestyle factors available within the dataset was created; fruit and vegetable consumption, body mass index, physical activity, smoking, alcohol consumption (Table 1) (Berstad et al., 2015; Helander et al., 2018). Details about the measures and methods have been described previously in a paper exploring determinants of interest in five topics of lifestyle advice in the context of cancer screening (Stevens et al., 2018). Higher index scores indicate a greater number of cancer protective factors reported by the participant.

The index variable was added to the three logistic regression models identifying correlates of interest in lifestyle advice at cervical, breast and bowel cancer screening (reported in main manuscript). The index was not associated with interest in lifestyle advice in any of the three screening scenarios, and did not influence the findings of the original models reported in the main manuscript (Table 2). However, due to missing data within the BMI variable (19.4% in cervical screening scenario; 20.4% in the breast screening scenario; 16.2% in the bowel screening scenario), the sample size for each model was reduced.

**References**

Berstad, P., Loberg, M., Larsen, I.K., Kalager, M., Holme, O., Botteri, E., Bretthauer, M., Hoff, G., 2015. Long-term lifestyle changes after colorectal cancer screening: randomised controlled trial. Gut 64:1268-76.

Helander, S., Heinavaara, S., Sarkeala, T., Malila, N., 2018. Lifestyle in population-based colorectal cancer screening over 2-year follow-up. Eur J Public Health 28:333-38.

Stevens, C., Vrinten, C., Smith, S.G., Waller, J., Beeken, R.J., 2018. Determinants of willingness to receive healthy lifestyle advice in the context of cancer screening. Brit J Cancer:119:251-275.

| **Lifestyle factor** | **Scoring** |
| --- | --- |
| Fruit and vegetable consumption | 1: Consumes five or more portions per day  0: Consumes less than five portions per day |
| Body mass index (BMI) | 1: BMI <25  0: BMI ≥25 |
| Physical activity | 1: Physically active for 30 minutes at least five times per week  0: Physically active for 30 minutes less than five times per week |
| Smoking | 1: Non smoker  0: Current or occasional smoker |
| Alcohol consumption | 1: Consumes < 14 alcoholic units per week  0: Consumes ≥ 14 alcoholic units per week |

**Table 1.** Scoring criteria for index of cancer protective factors

|  | Cervical screening sample (n=504)^1^ | | Breast screening sample (n=243)^1^ | | FS screening sample (n=188)^1,2^ | |
| --- | --- | --- | --- | --- | --- | --- |
|  | | | | | | |
|  | **OR** | **95% CI** | **OR** | **95% CI** | **OR** | **95% CI** |
| **Age** | 0.99 | 0.97-1.01 | 0.98 | 0.94-1.03 | 1.01 | 0.95-1.28 |
| **Gender** | | | | | | |
| Male | - | - | - | - | REF | **-** |
| Female | - | - | - | - | 2.73 | 1.17-6.34 |
| **Ethnicity^3^** | | | | | | |
| White | REF | - | REF^4^ | - | REF | - |
| Non-White | 2.97 | 1.15-7.65 | - | - |  |  |
| **Education** | | | | | | |
| Degree level or above | REF | - | REF | - | REF | - |
| Qualifications below bachelor’s degree level | 0.52 | 0.31-0.88 | 0.70 | 0.30-1.62 | 0.47 | 0.16-1.39 |
| **Cancer protective factor index** | 0.99 | 0.78-1.25 | 1.00 | 0.71-1.41 | 1.01 | 0.69-1.47 |
| ^1^Data is presented unweighted  ^2^ Flexible Sigmoidoscopy  ^3^Based on dichotomisation of UK Census classifications  ^4^ No estimate due to missing data | | | | | | |

**Table 2:** Correlates of willingness to receive lifestyle advice in cervical, breast and FS screening scenarios (adjusted logistic regression models)
